# Unveiling the Diagnostic Potential of Linguistic Markers in Identifying Individuals with Parkinson’s Disease through Artificial Intelligence: A Systematic Review

**DOI:** 10.3390/brainsci14020137

**Published:** 2024-01-28

**Authors:** Cinzia Palmirotta, Simona Aresta, Petronilla Battista, Serena Tagliente, Gianvito Lagravinese, Davide Mongelli, Christian Gelao, Pietro Fiore, Isabella Castiglioni, Brigida Minafra, Christian Salvatore

**Affiliations:** 1 Istituti Clinici Scientifici Maugeri IRCCS, Laboratory of Neuropsychology, Bari Institute, 70124 Bari, Italy; cinzia.palmirotta@icsmaugeri.it (C.P.); simona.aresta@icsmaugeri.it (S.A.); gianvito.lagravinese@icsmaugeri.it (G.L.); davide.mongelli@icsmaugeri.it (D.M.); 2 Istituti Clinici Scientifici Maugeri IRCCS, Neurorehabilitation Unit of Bari Institute, 70124 Bari, Italypietro.fiore@icsmaugeri.it (P.F.);; 3 Department of Physical and Rehabilitation Medicine, University of Foggia, 71122 Foggia, Italy; 4 Department of Physics G. Occhialini, University of Milan-Bicocca, 20133 Milan, Italy; 5 Department of Science, Technology and Society, University School for Advanced Studies IUSS Pavia, 27100 Pavia, Italy; christian.salvatore@iusspavia.it; 6 DeepTrace Technologies S.R.L., 20122 Milan, Italy

**Keywords:** PD, machine learning, automated classification, natural language processing, narrative speech, discourse

## Abstract

While extensive research has documented the cognitive changes associated with Parkinson’s disease (PD), a relatively small portion of the empirical literature investigated the language abilities of individuals with PD. Recently, artificial intelligence applied to linguistic data has shown promising results in predicting the clinical diagnosis of neurodegenerative disorders, but a deeper investigation of the current literature available on PD is lacking. This systematic review investigates the nature of language disorders in PD by assessing the contribution of machine learning (ML) to the classification of patients with PD. A total of 10 studies published between 2016 and 2023 were included in this review. Tasks used to elicit language were mainly structured or unstructured narrative discourse. Transcriptions were mostly analyzed using Natural Language Processing (NLP) techniques. The classification accuracy (%) ranged from 43 to 94, sensitivity (%) ranged from 8 to 95, specificity (%) ranged from 3 to 100, AUC (%) ranged from 32 to 97. The most frequent optimal linguistic measures were lexico-semantic (40%), followed by NLP-extracted features (26%) and morphological consistency features (20%). Artificial intelligence applied to linguistic markers provides valuable insights into PD. However, analyzing measures derived from narrative discourse can be time-consuming, and utilizing ML requires specialized expertise. Moving forward, it is important to focus on facilitating the integration of both narrative discourse analysis and artificial intelligence into clinical practice.

## 1. Introduction

Parkinson’s disease (PD) is a lifelong chronic progressive neurodegenerative disease primarily caused by a loss of dopaminergic neurons in the nigrostriatal pathway as a result of massive neuronal degeneration of the basal ganglia structures (e.g., substantia nigra pars compacta, striatum, putamen) [1,2]. The clinical picture of PD is characterized by motor (bradykinesia, rigidity, resting tremors, and postural instability) and non-motor symptoms, i.e., sleep disturbances, affective and behavioral disturbances, autonomic dysfunctions, cognitive deficits affecting executive functions, memory and learning, and visuospatial abilities [3]. In addition, speech and language disorders may be present, in particular, motor speech disorders affecting articulation and intelligibility of speech, such as hypokinetic dysarthria. However, language impairments are also commonly reported in almost 50% of patients [4,5], irrespective of changes in motor speech [6], cognitive decline, or motor symptoms [7]. This is not surprising considering that the main neural structures affected in PD are the frontostriatal networks (disruptions along basal ganglia–thalamo-cortical motor circuits), which play a crucial role in language formulation and processing [8,9,10].

Patients with PD can manifest both basic and complex language production difficulties [11], ranging from single-word levels such as lexical retrieval difficulties [12,13,14] and verbal fluency deficits, especially in those tasks involving action words or action fluency to narrative discourse production [15,16,17,18,19,20,21,22,23,24]. Concerning narrative discourse, patients with PD, although able to produce a similar number of words and well-organized phrases as control subjects, manifest fluency disruptions in various ways, such as incomplete utterances, extended breaks within or between sentences, or filled or unfilled pauses [25], that can be ascribed to challenges encountered in idea generation, preparation, and the initiation of discourse, as well as difficulties during motor programming and articulation [26]. There are also deficits in grammatical formulation, more than syntactical, and fewer contents of narrative speech [7,25,27].

Artificial intelligence has proven to be a new and effective way of supporting the analysis of multivariate complex and huge data, providing classifications of individual subjects. Machine learning (ML) is a field of artificial intelligence that exploits sophisticated computational methods capable of automatically categorizing subjects for diagnostic purposes [28]. In the field of neurodegenerative diseases, ML was applied first to support neuroimaging analysis [29] and biological data [30] and, more recently, to neuropsychological data [31,32], with high classification performances, especially in sensitivity/specificity. Diverse methodologies centered around speech signals and advanced ML frameworks were explored in PD, providing a user-friendly, contextually relevant, and unbiased framework for gathering clinically applicable data [33,34]. Although language has been less studied in PD patients, studies on the application of artificial intelligence to linguistic data are increasingly emerging, considering the evidence that supports the classification of patients with Alzheimer’s Disease when using language measures [35].

First, we aimed to analyze and summarize all studies reporting linguistic measures extracted with an ML approach for classification and clinical diagnosis in PD by reviewing studies comparing patients with PD to controls. Thus, we selected the included studies based on predetermined inclusion criteria, and then we highlighted and compared the main characteristics of these works, including the methodological approaches adopted. Given the fact that we expect some heterogeneity in language outcomes, the second aim of this study was to investigate which PD features were the optimal predictors in the classification of PD vs. controls. Finally, we discuss the importance of a better characterization of a language profile in these patients, which would contribute to a more fine-grained phenotyping, differential diagnosis, and tailored interventions in patients with PD.

## 2. Materials and Methods

### 2.1. Search Strategy and Selection Criteria

This systematic review was conducted on papers published on the use of ML applied to the automatic classification of PD through linguistic features. The protocol of this study was not registered. This work was performed and reported following Preferred Reporting Items for Systematic Reviews and Meta-Analyses (PRISMA) guidelines [36]. The PICOS approach was used to identify the studies to be included in the review. Criteria for including or excluding papers were determined a priori. Papers were considered for inclusion only if (a) they were written in full-text English language in a peer-reviewed journal; (b) they were published without a predetermined initiation date, allowing for inclusion of relevant literature published prior to our search on 27 November 2023; (c) they included subjects with a primary diagnosis of PD according to current clinical criteria of the UK Parkinson’s Disease Society Brain Bank [2]; and (d) they included linguistic measures for the classification. Articles were excluded if (a) linguistic measures were not included in the classification process, (b) there was no classification of patients with PD vs. healthy subjects (HC) performed, (c) they could not provide any classification performance, (d) they considered only non-linguistic tasks, (e) they considered only speech features, and (f) they considered subjects with a history of other neurological or psychiatric disorders such as Alzheimer’s Disease or atypical parkinsonism. Two authors screened the publications on their relevance for the review. The final resulting papers were considered eligible for review.

### 2.2. Information Source and Search

A comprehensive search strategy was designed with the assistance of a librarian, using a combination of keywords and medical subject headings (MeSH). Two of the authors (CP, SA) independently conducted an extensive literature search using the following databases: MEDLINE, CINAHL, and PsycINFO. The search was concluded on 27 November 2023. The search strategy based on the PICOS approach was applied following five concepts: (1) Patient, defined as subjects with PD; (2) Intervention, defined as the linguistic measures used as classifiers; (3) Comparison, defined as the clinical diagnosis of PD; (4) Outcome, defined as the predicted outcome, which was, for example, “discrimination of PD patient from healthy subjects”; and (5) Type of the study, which should be “longitudinal studies” or “nested case-control studies”. The search strategy was formed around three concepts: “PD”; “linguistic data” and “machine learning”. Synonyms within each concept were combined with OR Boolean operator, and terms between concepts were combined with AND Boolean operator. The following keywords (with both extended names and abbreviations) were used for the literature search: ((“Parkinson’s disease”) AND (“language assessment” OR “linguistic measures” OR “language test” OR “linguistic analysis” OR “linguistic features” OR “language”) AND (“machine learning” OR “deep learning” OR “artificial intelligence” OR “automated”)). In order to increase the likelihood that all the potentially relevant studies were identified, further papers were included by the two authors from a manual search, starting from the lists of references of previously retrieved articles.

### 2.3. Study Selection

The study selection was carried out by two reviewers (CP, SA), independently. The studies retrieved by the search strategy were first screened based on the titles and then selected by one of the two reviewers (CP) based on abstracts. One additional reviewer (PB) independently revised the list of potential articles based on abstracts. The articles considered to be potentially eligible were then evaluated in detail by the same reviewer for quality assessment, and any unresolved issues were discussed with CS. These papers were checked by studying the full text to exclude papers that did not meet inclusion criteria when this was not directly clear from the title and abstract. All articles reporting data that could be appropriately pooled were included in the quantitative analysis. Specifically, we restricted our analysis to those papers that reported at least one measure of the automatic classification performance among accuracy, sensitivity, specificity, Area Under the ROC Curve (AUC), precision, and F1-score.

### 2.4. Data Extraction Strategy

The data collected from each article were categorized as information on the first author and year of publication, the size of cohorts, the performed task, the methods used for linguistic analysis, the linguistic measures used as input for the ML algorithm clustered according to the linguistic level; the classification algorithm; the classification performance in terms of study-specific accuracy, study-specific specificity, study-specific sensitivity, study-specific AUC, study-specific precision, and study-specific F1-score; and optimal predictors extracted from the ML analysis grouped according to their linguistic level. To classify the optimal predictors, we considered linguistic levels, i.e., phonetic and phonological, lexico-semantic, morphosyntactic, and discourse and pragmatic, based on the role they play in the narrative speech, respectively, sound and/or phoneme, word, sentence, or discourse. Features related to the age at which a word and its meaning were first learned or the property of a word to elicit a mental image, visual representation, or other sensory experience (psycholinguistic) were classified as lexico-semantic. However, some of the extracted predictors cannot be included at a specific linguistic level as they are engineering features derived from deep learning analysis methods: from here on, we will refer to them as NLP-extracted features. These are word embeddings that capture both semantic and syntactic word information by placing vectors of words similar in meaning closer together in a vector space. Also, sentiment scores deriving from NLP sentiment analysis cannot be classified into one of the linguistic levels, as they refer to the emotional tone of a text (e.g., sentiment). Finally, some features refer to statistical functions calculated on Part-of-the-Speech tag probability scores, which indicate the estimated probability that a part of speech has a specific attribute and value (morphological consistency). Table 1 summarizes and reports a definition of each defined level.

### 2.5. Risk of Bias in Individual Studies

Following the Cochrane guidelines, the Quality Assessment of Diagnostic Accuracy Studies (QUADAS) tool [37] was used to assess the methodological quality and the risk of bias of each study. This quality assessment allowed classifying studies as having low, high, or unknown risk of bias. We used a high-quality report subgroup for meta-analyses.

## 3. Results

### 3.1. Study Selection

**Figure 1:**
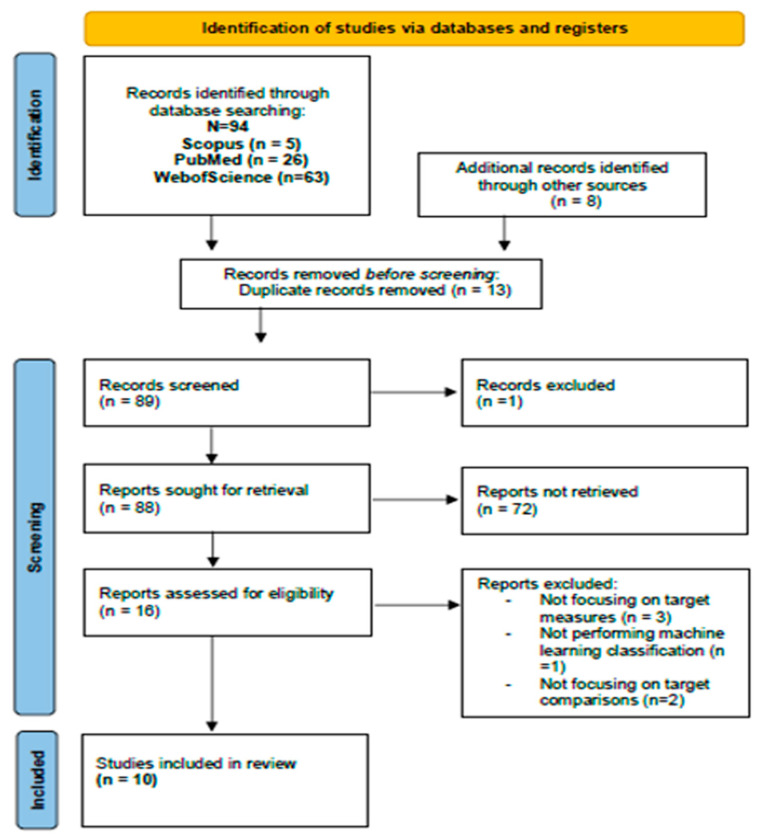
PRISMA flow diagram depicting the different phases of the review selection process.

All the phases of the selection process are shown in the PRISMA flow diagram in Figure 1. The literature search yielded 94 papers from electronic databases, and eight more papers were included in the reference list of previously retrieved articles. A total of 102 papers were identified. Moreover, 13 papers were removed before screening because they were duplicates. A total of 89 papers were screened. One document was excluded because it was an abstract from a meeting and relevant information was missing, which left 88 records. Another 72 papers were further excluded because (a) they focused on a different topic, (b) automatic classification was not performed, (c) different populations were investigated, (d) no linguistic measures were provided. At this point in the screening, there were 16 papers left. At this step, three papers were excluded because they did not provide linguistic measures; one paper was excluded because it did not perform an ML classification; two papers were excluded because the control group was missing, and the comparison was made within the same group of patients with PD in two different medication states or with other neurodegenerative disorders (e.g., Progressive Supranuclear Palsy). Finally, 10 papers were selected as eligible, which were, therefore, included in this review (Figure 1).

### 3.2. Study Characteristics

Table 2 shows papers included in the systematic review and reports the sample size, categories (e.g., PD vs. HC), the linguistic task employed in the study, the linguistic analysis method, the linguistic measures used as input to train the ML algorithm adopted, the performance measures (in terms of accuracy, sensitivity, specificity, Area Under the ROC Curve (AUC), precision, and F1-score), and linguistic features extracted from the ML algorithm as optimal predictors for the classification task.

The final search retrieved 10 papers. The majority of papers (9 out of 10) focused their analysis on the comparison between PD and HC, one of which also differentiated between healthy younger subjects and healthy older subjects. One paper made a distinction between Mild Cognitive Impairment (MCI) and PD without MCI [47].

Concerning the other studies, the comparison was made between PD and HC; the median (range) of the cohort size was 51 (20–88) for patients with PD and 50 (16–88) for HC. Notably, one study did not report the sample size for patients with PD [46]. In these papers, cohorts included native speakers of different languages: Japanese, Colombian and Castilian Spanish, British and American English, German, and Czech.

In the selected studies, linguistic measures used to train the ML model were extracted from the transcriptions of spontaneous speech, connected speech, and retelling tasks and analyzed using statistical and word-embedding techniques. More specifically, in most cases, participants were asked to talk about their daily routine [25,38,39,40,43,44,45]. Other studies used semi-structured interviews where subjects were questioned about their favorite food and their interests, jobs, and families to elicitate spontaneous discourse [38,41]. A study conducted by Jessiman and colleagues [46] used two different tasks: in the first, participants were asked to produce scripts for 10 selected everyday activities, and in the second, participants were asked to provide directions to arrive at 5 familiar destinations. The study by Favaro et al. (2023) [41] reported results from the connected speech “Cookie Theft” picture description task. Only Garcìa and colleagues [42] developed ad hoc linguistic tasks to assess the presence of specific language disorders related to verbs, namely the “action” text task that assesses verbs related to the characters’ bodily movements and the “non-action” text that focuses on verbs expressing characters’ feelings, thoughts, and perceptions. Participants were asked to read the texts silently and to retell the story in their own words.

Regarding the linguistic method analysis employed by the included studies, transcriptions were analyzed by Natural Language Processing (NLP) methods. NLP refers to a branch of artificial intelligence that makes a computer capable of analyzing, representing, and then understanding text and spoken words in the same way that humans do [48,49]. NLP combines computational linguistics with statistical, ML, and deep learning models. In the selected studies, several NPL methods were used: (1) tokenization and part-of-speech tagging, consisting of splitting sentences or words into semantically useful units called “tokens” and giving each token a label indicating its speech category [25,38,41,43,44,45,46]; (2) dependency parsing, used to analyze the grammatical structure in a sentence and define the relationships between words [38,46]; (3) named entity recognition, employed to detect and categorize important information in texts; (4) semantic similarity between sentences, used to exploit word embeddings, that are representations of words in the form of numerical vectors capable of capturing context-dependent linguistic similarities [25,40,42,44]; (5) sentiment analysis used to identify positive or negative emotions in a text [46].

The most frequently used ML algorithm for automated classification was the Support Vector Machine (SVM), used in 8 out of 10 papers [25,38,40,41,42,43,44,45]. Other ML algorithms frequently used were Random Forest (RF) [41,44,46], 1D-Convolutional Neural Networks (1D-CNN) with fully connected layer [39,40], Logistic Regression (LR) and Stochastic Gradient Descent (SGD) [43], K-nearest neighbors (KNN) [25,41], Boosting (BG) and Extreme Gradient Boosting (XGBoost) [41], Ada Boost, Linear Discriminant Analysis (LDA), and Naive Bayes (NB) [25].

Furthermore, within the same study, multiple models were compared to identify the best classifier chosen based on its performance, in 4 out of 10 papers [25,38,42,43] identified the SVM as the best model, 1D-CNN [39,40], RF [43,44], XGBoost [41], LR [43], SGD [43], and KNN [25].

### 3.3. Risk of Bias within Studies

**Figure 2:**
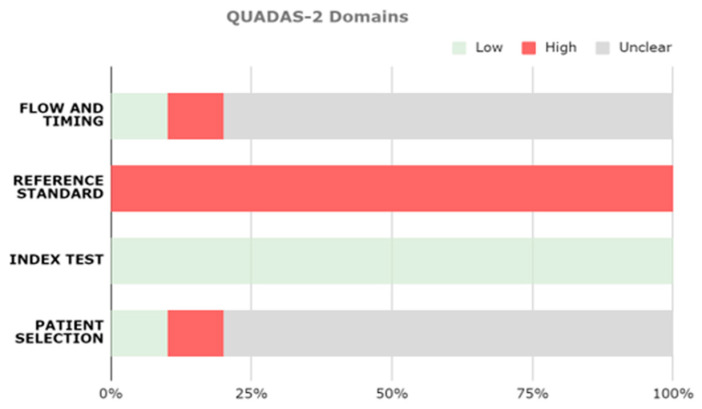
Proportion of studies with low, high, or unclear risk of bias.

The risk of bias associated with studies, as well as the comments of the authors concerning the seven domains of the QUADAS tool, was assessed. Figure 2 shows the QUADAS-2 domains assessed for the studies included in the review. All studies achieved low concerns regarding applicability because the characteristics of patients, the setting, the conduction and interpretation of the index test, and the target condition as defined by the reference standard matched the review question.

Regarding the assessed domains, all studies presented a high risk of bias about the appropriateness of the reference standard (e.g., none of the included studies used neuropathological data as their gold standard for the diagnosis); most of them did not provide enough information about patient selection (6/10) and flow and timing (8/10). Since measures used as the gold standard for the diagnosis were independent of measures used as input for ML algorithms’ training, all studies achieved a low risk of the appropriateness of the index test.

The risk of bias tools highlighted the following limitations:

- There was no mention of post-mortem analysis for diagnosis confirmation in any of the studies;
- Only two studies included sufficient details about the selection process and reported that inappropriate exclusions were avoided [38,43]. Concerning sample enrollment, in one study, part of the HC subjects were recruited via convenience sampling [46], while in another paper, the authors did not specify the database they used for the study [40];
- Only one study mentioned that all participants recruited were included in the analysis [43].

### 3.4. Results of the Systematic Review

**Figure 3:**
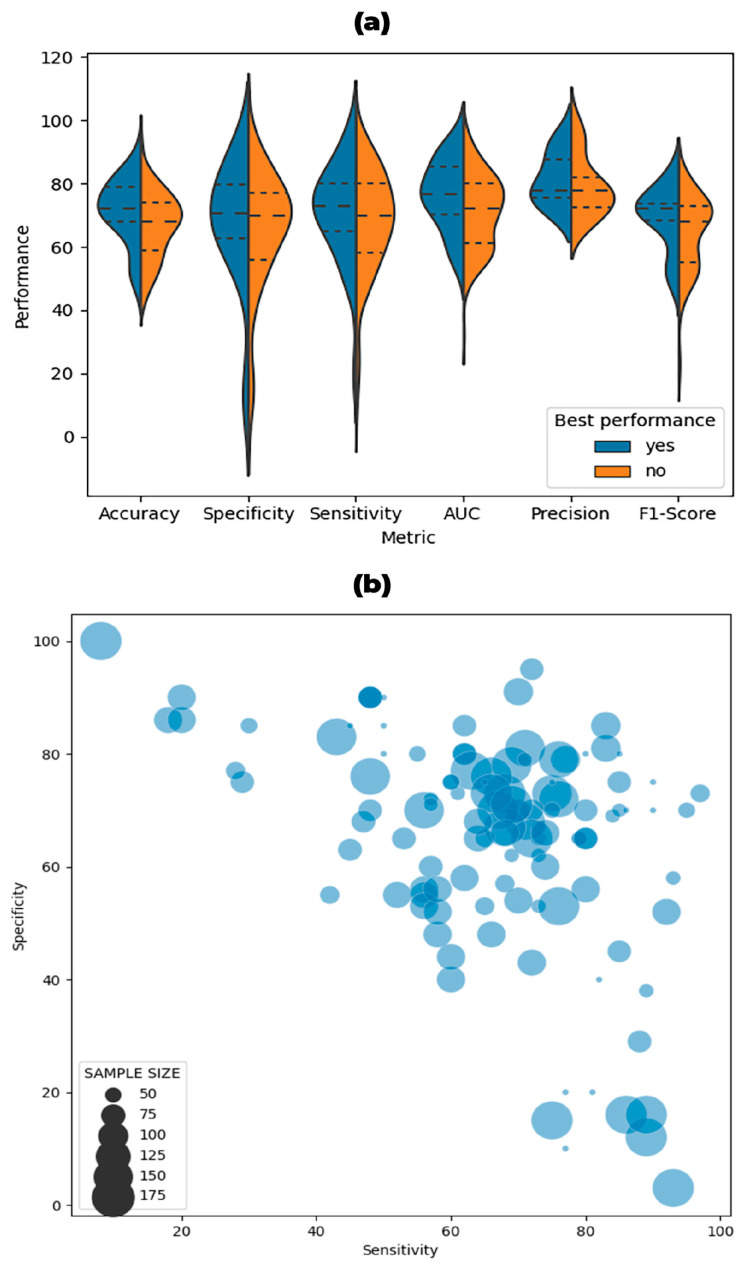
(**a**). Violin plots to graphically show the performance in terms of Accuracy, Sensitivity, Specificity, AUC, Precision, and F1-Score for the comparison of PD vs. HC. The violin plots show the distributions of these metrics when considering all the performances reported by the papers (right side of the violin plots in red) or only the best performances highlighted by the authors (left side of the violin plot in blue). When the authors did not indicate which of the reported models was considered the best, the choice was made by the authors of the present systematic review based on AUC. The means and quartiles of the performance distributions are also reported as dotted lines. (**b**). Bubble chart showing the distribution of the models for sensitivity and specificity (when reported). The best models are in the upper right corner, which have high sensitivity and specificity. Models with high specificity and low sensitivity are in the upper left, whereas models with high sensitivity and low specificity are in the bottom right. The size of the points is proportional to the sample size used to train, validate, and test the models, as reported in the considered papers.

The results of our systematic review were obtained from papers grouped in Table 2. The violin plots in Figure 3a graphically show performance results regarding Accuracy, Sensitivity, Specificity, AUC, Precision, and F1-score in PD-vs-HC classification. In Figure 3b, the bubble chart displays model distribution according to sensitivity and specificity performance metrics with respect to the proportional sample size dimension.

**Figure 4:**
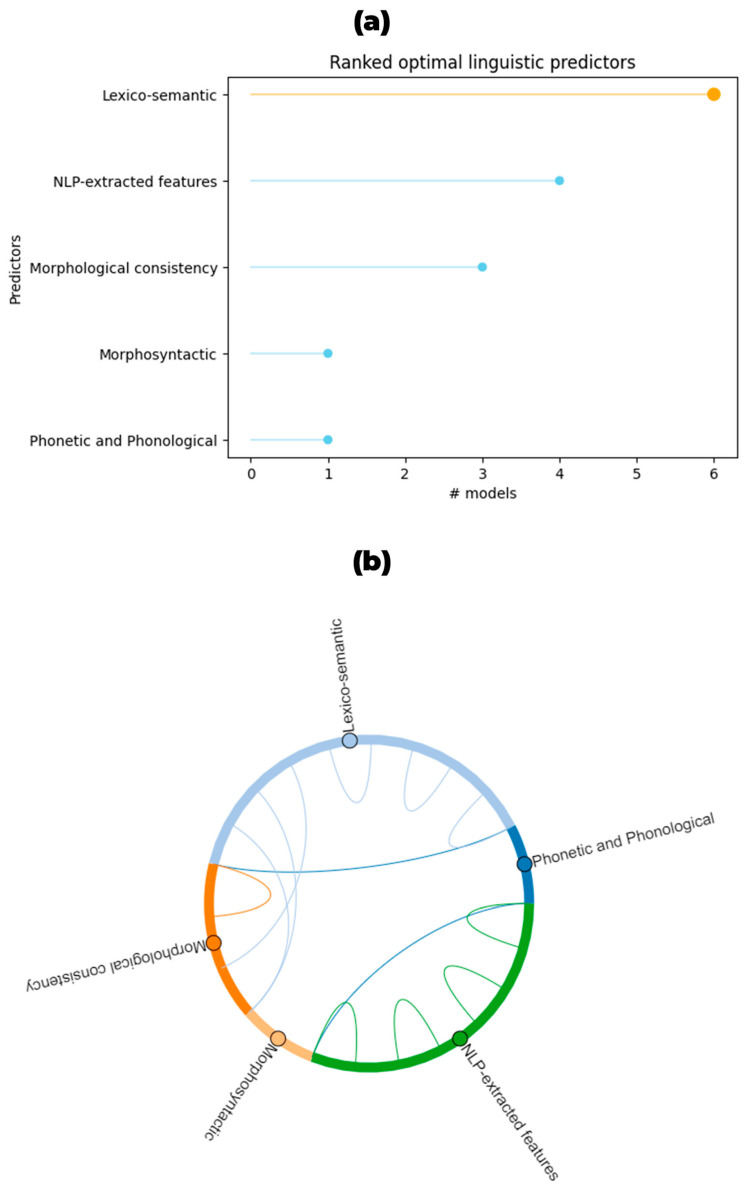
(**a**). Lollipop plot for the optimal predictors for the comparison of PD vs. HC. (**b**). Chord diagram showing the connections between optimal features in the considered models (when reported). The plot shows how many times optimal features are associated with each other.

Furthermore, the optimal predictors identified in the classification task under study are graphically summarized in the lollipop plot shown in Figure 4a, whereas the chord diagram in Figure 4b shows the connection between optimal predictors when reported in the considered models.

As shown in Figure 3a, focusing on the selected papers, the classification accuracy (%) ranged from 43 to 94, sensitivity (%) from 8 to 95, specificity (%) from 3 to 100, AUC (%) from 32 to 97, and F1-scores (%) from 21 to 85. The best classifiers’ accuracy (%) ranged from 49 to 89, sensitivity (%) from 20 to 90, specificity from 10 to 90, AUC from 55 to 94, precision from 73 to 94, and F1-score from 49 to 82. Figure 3b highlights that most of the models reported in the selected papers presented either high sensitivity or specificity, and only a few presented a Specificity higher than Sensitivity or the opposite, without any influence of the sample size used in the training, validation, or testing phase.

Garcìa and colleagues [42], as previously reported, besides performing the PD vs. HC classification task, wanted to highlight linguistic profile differences between PD-MCI vs. HC and PD-nMCI vs. PD-MCI. In PD-MCI vs. HC, the classification accuracy (%) ranged from 47 to 85, specificity (%) from 29 to 85, sensitivity (%) from 28 to 97, AUC (%) from 49 to 93, and F1-score from 42 to 84. When comparing PD-MCI vs. PD-nMCI, the classification accuracy (%) ranged from 59 to 69, specificity (%) from 63 to 90, sensitivity (%) from 20 to 75, AUC (%) from 53 to 82, and F1-score from 44 to 67. In contrast, Jessiman and colleagues [46], beyond performing the binary PD vs. HC classification, subdivided HCs in young (HYA) and older adults (HOA) according to their age, respectively, 27.2 years and 69.1 years, and found that the HYA vs. HOA vs. PD classification accuracy (%) was 63 at the participant level and 59 at the document level. Moreover, two studies considered either patients with PD or HC subjects with different native spoken languages [41,43]. Specifically, in Favaro and colleagues [41], there were American English, Castilian Spanish, Colombian Spanish, German, and Czech PD and HC populations. The best PD vs. HC accuracy classification was achieved in the Castilian Spanish population using the XGBoost model (85%), whereas the lowest was in the Czech population (43%). In Eyigoz and colleagues [43], there were Spanish, German, and Czech PD vs. HC populations. The best PD vs. HC accuracy classification was achieved in the Czech population (94%), whereas the lowest was in the Spanish one (65%). There was much heterogeneity among the linguistic measures used in the studies; thus, it was useful to group them according to a specific linguistic category. Linguistic measures extracted from transcriptions through NLP methods and used to train ML models, as well as those selected as optimal predictors for the ML classification task, are shown in detail in Table S1 of the Supplementary Materials.

The linguistic measures most frequently used as input for the classification and with good overall accuracy were lexico-semantics, such as different words or parts of speech frequency [25,41,44,45,46], different parts of speech ratio or type/token ratio [38,43,46], and NLP-Extracted features [25,39,40,42,44,46]. Other frequently used measures belong to the morphosyntactic level (e.g., number of sentences, dependency distance, and sentence length [38,41,43,46]). The majority of the included papers identified features with greater discriminatory power in PD vs. HC classification [25,38,39,40,42,43,44,45]. In Figure 4a, the ranking of the optimal predictors found is shown; the most frequent (≥25% frequency) were lexico-semantic features and NLP-extracted features. Lexico-semantic features included subordinating conjunctions [25,43], verb ratio and verb utterance ratio [38], proper nouns and proper noun utterance ratio [38,43], personal pronouns [43], general noun utterance ratio [38], negative markers [25], words frequency [44], and morpheme prefixes [45]. NLP extracted features were verb embeddings [39,42], word embeddings [40], and semantic components [25]. Three ML models found morphological consistency to have good discriminating power [43]. This study included proper nouns skewness and present tense verbs mean for Spanish; neuter gender pronouns kurtosis, verb person not specified skewness, determiner in accusative case skewness, feminine nouns standard deviation for German, and person not specified skewness, 2nd most frequent variant kurtosis, and masculine gender skewness for Czech. One model identified morphosyntactic features as optimal predictors, in particular, the case particle ratio dispersion [38]. Only one model highlighted the filler utterance ratio among the optimal predictors, a measure that belongs to the phonetic and phonological levels [38]. In a few other studies, the optimal predictors were not reported, as the authors aimed to identify the best methodological combination consisting of an ML model and a linguistic representation method, which better discriminates PD from HC [41,46].

Lastly, when exploring the connections between optimal predictors, NLP-extracted features mostly occur alone as optimal predictors (4 out of 11 considered models reporting the optimal predictors), as well as lexico-semantic features (3 out of 11); the association between lexico-semantic and morphological-consistency features as optimal predictors occurs two times (Figure 4b).

## 4. Discussion

The aim of this review was to examine the existing literature rregarding the ML contribution and language measures in classifying patients with PD and to report the results of selected studies in terms of optimal predictors in discriminating PD from HC, as well as sample characteristics, methodologies used for feature extraction and classification task, and performance of ML algorithms. For this purpose, we reviewed 10 studies published between 2016 and 2023, which made a comparison between PD and HC using different ML algorithms.

Most of the models obtained in the different studies presented good performance, with high sensitivity/specificity besides accuracy; the best classifiers achieved an accuracy of up to 89% and were found to have good performances in identifying both true positives and true negatives (Sensitivity = 90%, Specificity = 90%), with no models presenting low sensitivity and specificity. Only a few studies presented greater sensitivity than specificity, thus predicting PD patients better than HC subjects or greater specificity than sensitivity, predicting HC subjects better than PD patients. In addition, the distribution of the sample size used to train, validate, and test appears to be homogeneous, i.e., there were no particular differences in performance between models with higher vs. lower sample sizes (as displayed in Figure 3b).

Despite previous studies focusing their attention mainly on phonatory and articulatory deficits in patients with PD and training ML algorithms on acoustic features extracted from speech signals [34,50], our results show that linguistic features extracted from spontaneous and connected speech transcriptions are useful in the automatic diagnosis of PD regardless of the language spoken by the recruited samples and the different ML algorithms. Furthermore, subsets of optimal classification features were selected as predictors able to characterize PD and HC in most of the papers considered in our review. Great heterogeneity can be seen among features that emerged from ML analyses. This may be partly because language can be investigated at different levels, and the included studies focused on a different set of measures. Moreover, languages involved in the selected studies were structurally dissimilar; thus, lexico-semantic and morphological features specifically varied considerably across languages.

The most relevant predictors in classifying PD vs. HC were lexico-semantic features. In particular, we found verb ratio, general noun utterance ratio, proper noun utterance ratio, verb utterance ratio, subordinating conjunctions, proper nouns, personal pronouns, negative markers, word frequency, and prefix-related features (i.e., prefix–prefix pair, prefix length probability, prefixes probability, prefix–stem probability). There is considerable evidence suggesting that, even in the absence of dementia, the ability to process lexico-semantic aspects of language is impaired in PD. These findings align with previous evidence that demonstrates the tendency of patients with PD to exhibit less informative narratives. In individuals with PD, there is a reported notable decrease in the production of conceptual units, a lower presence of informative elements, and a decrease in semantic abilities [8,51]. The presence of less well-formed words and fewer concept units, irrespective of articulation rate, suggests that individuals with PD experience challenges in linguistic processing. Specifically, this difficulty lies in the ability to access and retrieve target words from their mental lexicon, thereby affecting overall productivity in language expression [52].

Among the different measures identified as optimal predictors, features extracted from NLP (verb embeddings, word embeddings, and semantic components) provided a greater contribution to the classification, opening new pathways to the study of the language profile of PD. These features capture morphosyntactic and semantic properties, and relationships between words using artificial intelligence methods, which are able to directly process text and extract features that cannot be detected by other methods. In the last few decades, NLP methods have already been proven to be useful for the assessment of neurological and mood disorders, as they are able to capture early biomarkers of the disease that are not detectable using conventional clinical methods, and they can be applied to a wide range of subjects considering their low costs [53]. A previous study by Beltrami and colleagues [54] showed how lexical and syntactic features, extracted from connected and spontaneous speech transcriptions using NLP techniques, were essential to identify early signs of MCI in an elderly population with a higher discriminatory power than traditional neuropsychological tests, including tests designed to assess phonological and semantic fluency, as they are not influenced by the subjective component introduced by the clinicians analyzing the transcript, being directly obtained from the NLP model. Although NLP features are not easily interpretable from a clinical point of view and not classifiable at a specific linguistic level, they are becoming widely used also in clinical studies given their potential. NLP features are directly extracted from the processed text by the model itself in the feature engineering step and, at the same time, learned by the model in the feature learning one. Other studies showed the contribution of linguistic features, particularly semantic features, extracted from connected speech transcriptions through NLP methods to detect dementia [55] and word-embedding from their spontaneous speech to explore depressive patterns in patients with PD [56]. Furthermore, word embeddings derived from social media texts proved to be useful for performing sentiment analysis to easily develop a personal care plan [57]. Therefore, NLP is able to interpret, understand, and utilize human language with several advantages that lie in its ability to enable human-like language processing and perform a multitude of tasks by analyzing and representing naturally occurring texts at different levels of linguistic analysis. However, it is important to consider the potential disadvantages that may arise from the complexity of NLP tasks and the challenges in achieving complete accuracy and understanding in language processing [58]. For instance, human speech often relies on contextual cues; thus, a computer trained to search for specific words may not necessarily understand a specific context. Further, human language exhibits irregularities, such as variations within the same language, which can result in a lack of context, spelling errors, or differences in dialects. These irregularities further complicate the interpretation of NLP features, and future studies are needed to solve these issues.

Moreover, among the lexico-semantic and the NLP-extracted levels, we found verb-related features frequently in discriminating PD from HC, consistent with the previous literature that reported a selective impairment of action verbs with relative preservation of nouns showing a dissociation between verbs and nouns in patients’ performances in different linguistic tasks in patients with PD [59,60,61]. Verbs may involve a higher cognitive effort than other grammatical classes because they require the selection of a specific verb form from a wide range of words that share the same verb root [62]. Considering that the impairment of the corticostriatal circuits connecting the striatum to the dorsal frontal cortex, including the inferior frontal gyrus, is a hallmark of PD, a deficit in verb production can be expected in this population. In fact, this extensive neural network supports verb processing (comprehension and production), and basal ganglia, together with the inferior frontal gyrus, regulate selection processes. Therefore, a deficit in verb production can be interpreted as a loss of executive control [63]; as suggested by current cognitive–linguistic models [64], there is a dynamic relationship between the linguistic component and the executive component of narrative production. The linguistic component primarily focuses on organizing information at the sentence level, while the executive resource component contributes to the organization of narratives at the discourse level. Given the high occurrence of executive deficits in PD and the cognitive demands involved in planning, initiating, and sustaining narrative discourse, changes in verb production can be explained as the consequence of this cognitive mechanism. In line with previous studies that found distinctive deficits in processing action language in people with motor diseases [22,65], some of the included papers highlighted the emergence of semantic fields related to actions and physical movements. Since the striatum receives connections from the motor cortex and takes part in movement control, a deficit in verb production has also been interpreted as an impairment in the semantic representation of verbs, according to the hypothesis that motor information is part of word meaning, and it is deteriorated in the presence of damages to neural structures involved in motor functions [66]. It is important to note that these findings may be influenced by the specific task employed by the researchers, as participants were required to describe their daily routines; however, individuals with PD typically demonstrate reduced engagement in motor-related activities compared to controls.

Concerning the limitations presented by the included studies, we found that the diagnostic reference standard was, most of the time, not appropriate, as often there is no mention of neuropathological data for the diagnosis confirmation. Moreover, some of the included studies did not provide enough information about patient selection and possible inappropriate exclusions of subjects. To judge the quality of the included studies, we decided to employ the QUADAS tool; however, it must be underlined that some of the items included in the QUADAS tool were not fully applicable to studies adopting ML approaches. On the other hand, the presence of bias in ML studies often arises from the data preparation process for training models. For instance, if a dataset is not divided into separate training and testing subsets, the model will be trained and evaluated on the same data. While this may yield impressive results (this potential hazard is commonly referred to as “overfitting”), there is a high probability that the model’s performance will significantly decline when tested on new, unseen data.

This systematic review also highlights some limitations which could guide future research in this field. The first limitation concerns the relatively small number of studies included. Our review reveals that the automated classification of PD is becoming more popular; still, relatively few studies have explored the diagnostic role of linguistic features. Moreover, most research in PD focused on acoustic features of speech rather than on cognitive components of language. It must be noted that during the selection process, many studies were excluded because they did not include a comparison group of controls. The second main limitation is related to the assessment of the cognitive status of participants; indeed, the included studies often did not consider whether patients presented with cognitive impairments. Only one paper differentiated PD-MCI from PD-nMCI and HC [42], and one paper reported as an inclusion criterion a MOCA score higher than 23, thus including patients with global cognitive efficiency within the normal range [38]. Although, in several studies, PD-related language deficits were found to be specifically isolated features independent of the global cognitive status [67], the fact that language is a component of cognition cannot be ignored. Thus, specific features that appear to be optimal predictors for PD classification could be related to the degree of cognitive impairment and not directly from disease-related impairment. In addition, the pharmacological therapy in the selected studies is not always reported, and fluctuations due to “ON” and “OFF” states induced by levodopa intake can affect language disorders in PD [68].

Finally, nearly all the studies included in this review report promising results; however, a small number of tools were put into clinical practice. We made a previous tentative with a digital tool to test whether touch screening devices could be ecological for clinicians [69]; more recently, Garcia and colleagues developed a new device, namely the Toolkit to Examine Lifelike Language (TELL), to capture linguistic markers of neurodegenerative disorders through automated speech and language analysis [70]. Although we expect an increase in studies that use ML to classify PD based on linguistic measures, the primary limitations that hinder progress in this field include inadequate standardization and clinical validation, limited comparability of results, and a gap between the objectives of the studies and their clinical applications. Efforts are being made to address these limitations, as artificial intelligence tools are less expensive and more accessible compared to other neuroimaging biomarkers that are not always available in the clinical setting and will help to bridge the gap between future research and its practical implementation in clinical settings. Artificial intelligence applied to linguistic data has the potential to enhance the treatment of PD in (1) *early diagnosis* by identifying subtle linguistic changes associated with the disease at an early stage, allowing a prompt intervention; (2) *objective assessment* providing quantitative measures of language and speech quality, pitch, loudness, and articulation, providing clinicians with valuable information for assessing disease progression and treatment effectiveness; (3) *personalized speech and language rehabilitation* by addressing individual needs and providing immediate feedback and guidance to patients with PD as they exercise, potentially accelerating their progress and improving overall speech outcomes; (4) *remote monitoring* by tracking changes in speech patterns over time, allowing healthcare providers to remotely assess disease progression and adjust treatment plans accordingly. This can reduce the need for frequent in-person visits and improve accessibility to care.

## 5. Conclusions

We examined the literature concerning the automated classification of PD using linguistic measures derived mainly from narrative discourse. Although linguistic data analyzed through artificial intelligence show promise in (1) the early diagnosis of PD by identifying a combination of optimal linguistic predictors and (2) the treatment addressed by shaping a tailored intervention, the clinical application of such tools still strongly needs the expertise of professionals from the healthcare field. The collaboration between artificial intelligence technology and medical professionals can lead to more effective and personalized care for individuals with PD.

## Figures and Tables

**Table 1:** Definition of the linguistic levels and other linguistic categories specified in the present study.

Specified Level	Definition
Phonetic and Phonological	Language production concerning speech sound aspects, i.e., number of pauses, total phonation time.
Lexico-semantic	Difficulties in words, classified according to different part-of-speech categories and content, i.e., verbs ratio, type/token ratio
Morphosyntactic	Information regarding word inflection and agreement encompassing elements like tense, mood, aspect, person, number, and gender, but also structure and word organization to form grammatically correct sentences and utterances, i.e., (number of utterances, correct verb tense.
Discourse and Pragmatic	Elements in the speech that contribute to the ongoing conversation flow, i.e., cohesion, total words.
NLP-extracted features	Engineered linguistic features derived from deep learning analysis methods, i.e., word embeddings, verb embeddings,
Morphological consistency	The estimated probability that a part of speech has a specific attribute and value, i.e., feminine nouns standard deviation, masculine pronouns skewness
Sentiment	Emotional text inflexion

**Table 2:** Overview of included studies.

Author (Year)	Comparison	Cohort	Task	Linguistic Analysis Method	NLP Input Features	ML Method	Acc/Spe/Sen (Recall)/AUC /Precision/F1-Score	Optimal Predictors
Yokoi et al. [38]	PD vs. HC	53 PD 53 HC	Spontaneous speech	MeCab Cabocha	Phonetic and Phonological Lexico-semantic Morphosyntactic	SVM	(1st trial) -/0.79/0.77/-/-/- (2nd trial) -/0.81/0.83/-/-/- **(3rd trial) -/0.85/0.83/0.88/-/-** (4th trial) -/0.91/0.70/-/-/-	Phonetic and Phonological: filler utterance ratio Lexico-semantic: verb ratio, general noun utterance ratio, proper noun utterance ratio, verb utterance ratio. Morphosyntactic: case particle ratio (dispersion)
Escobar-Grisales et al. (a) [39]	PD vs. HC	80 PD 85 HC	Spontaneous speech	BETO	NLP-extracted features	1D-CNN + FL	**(Verbs) 0.76/0.81/0.71/-/-/0.73**	NLP-extracted features: verb embeddings
							(Nouns) 0.73/0.78/0.69/-/-/0.70	
							(Statistical Functionals) 0.63/0.83/0.43/-/-/0.52	
Escobar- Grisales et al. (b) [40]	PD vs. HC	80 PD 85 HC	Spontaneous speech	W2V	NLP-extracted features	SVM 1D-CNN + FL	(SVM) 0.70/0.68/0.71/-/-/0.69 (CNN) 0.74/0.73/0.75/-/-/0.72	NLP-extracted features: word embeddings
				BERT			(SVM) 0.62/0.76/0.48/-/-/0.53 (CNN) 0.74/0.72/0.76/-/-/0.74	
				BETO			(SVM) 0.63/0.70/0.56/-/-/0.57 **(CNN) 0.78/0.79/0.76/-/-/0.77**	
Favaro et al. [41]	PD vs. HC (American English)	23 PD 27 HC	Connected speech (Cookie theft)	Whisper + SpaCy	Lexico-semantic Morphosyntactic Discourse and Pragmatic	SVM KNN RF BG XGBoost	(SVM) (mono-lingual) 0.76/0.69/0.84/0.82/-/0.73 (multi-lingual) 0.80/0.79/0.71/0.83/-/0.82 (cross-lingual) 0.67/0.73/0.61/0.70/-/0.68	n/a
							(KNN) (mono-lingual) 0.63/0.53/0.73/0.57/-/0.61 (multi-lingual) 0.73/0.70/0.85/0.80/-/0.71 (cross-lingual) 0.65/0.38/0.89/0.71/-/0.65	
							(RF) (mono-lingual) 0.70/0.67/0.73/0.80/-/0.70 (multi-lingual) 0.64/0.72/0.57/0.77/-/0.67 (cross-lingual) 0.76/0.58/0.93/0.74/-/0.70	
							(BG) (mono-lingual) 0.68/0.65/0.73/0.78/-/0.69 (multi-lingual) 0.68/0.71/0.57/0.76/-/0.70 (cross-lingual) 0.76/0.65/0.79/0.76/-/0.76	
							**(XGBoost)** ** (mono-lingual) 0.74/0.62/0.73/0.80/-/0.73** ** (multi-lingual) 0.68/0.62/0.69/0.77/-/0.70** ** (cross-lingual) 0.72/0.65/0.79/0.75/-/0.72**	
Favaro et al. [41]	PD vs. HC (Castilian spanish	32 PD 47 HC	Connected speech.	Whisper + SpaCy	Lexico-semantic Morphosyntactic Discourse and Pragmatic	SVM KNN RF BG XGBoost	(SVM) (mono-lingual) 0.85/0.75/0.85/0.82/-/0.82 (multi-lingual) 0.76/0.80/0.62/0.92/-/0.78 (cross-lingual) 0.51/0.75/0.29/0.78/-/0.60	n/a
							(KNN) (mono-lingual) 0.74/0.45/0.85/0.83/-/0.74 (multi-lingual) 0.68/0.70/0.72/0.82/-/0.68 (cross-lingual) 0.59/0.70/0.48/0.66/-/0.59	
							(RF) (mono-lingual) 0.76/0.70/0.70/0.81/-/0.73 (multi-lingual) 0.84/0.95/0.72/0.88/-/0.85 (cross-lingual) 0.68/0.90/0.48/0.85/-/0.73	
							(BG) (mono-lingual) 0.74/0.70/0.80/0.88/-/0.74 (multi-lingual) 0.74/0.85/0.62/0.91/-/0.74 (cross-lingual) 0.68/0.90/0.48/0.81/-/0.68	
							**(XGBoost)** **(mono-lingual) 0.71/0.65/0.80/0.88/-/0.71** **(multi-lingual) 0.74/0.80/0.62/0.86/-/0.74** **(cross-lingual) 0.68/0.90/0.48/0.79/-/0.68**	
Favaro et al. [41]	PD vs. HC (Colombian Spanish)	50 PD 50 HC	Spontaneous speech			SVM KNN RF BG XGBoost	(SVM) (mono-lingual) 0.51/0.40/0.60/0.50-/0.50 (multi-lingual) 0.52/0.44/0.60/0.56/-/0.51 (cross-lingual) 0.56/0.56/0.56/0.56/-/0.56	n/a
							(KNN) (mono-lingual) 0.54/0.65/0.64/0.57/-/0.54 (multi-lingual) 0.57/0.43/0.72/0.61/-/0.57 (cross-lingual) 0.56/0.56/0.80/0.32/-/0.52	
							(RF) (mono-lingual) 0.55/0.55/0.52/0.57/-/0.51 (multi-lingual) 0.58/0.48/0.66/0.60/-/0.53 (cross-lingual) 0.52/0.86/0.18/0.59/-/0.64	
							(BG) (mono-lingual) 0.56/0.55/0.56/0.60/-/0.56 (multi-lingual) 0.59/0.48/0.58/0.62/-/0.59 (cross-lingual) 0.53/0.86/0.20/0.58/-/0.53	
							**(XGBoost)** ** (mono-lingual) 0.55/0.53/0.56/0.57/-/0.55** ** (multi-lingual) 0.60/0.52/0.58/0.62/-/0.60** ** (cross-lingual) 0.51/0.90/0.20/0.55/-/0.51**	
Favaro et al. [41]	PD vs. HC (German)	88 PD 88 HC	Spontaneous speech	Whisper + SpaCy	Lexico-semantic Morphosyntactic Discourse and Pragmatic	SVM KNN RF BG XGBoost	(SVM) (mono-lingual) 0.57/1.00/0.08/0.57/-/0.80 (multi-lingual) 0.71/0.65/0.72/0.75/-/0.69 (cross-lingual) 0.45/0.15/0.75/0.52/-/0.21	n/a
							(KNN) (mono-lingual) 0.73/0.77/0.63/0.75/-/0.73 (multi-lingual) 0.66/0.53/0.76/0.71/-/0.66 (cross-lingual) 0.48/0.03/0.93/0.55/-/0.48	
							(RF) (mono-lingual) 0.69/0.73/0.68/0.72/-/0.70 (multi-lingual) 0.68/0.67/0.68/0.74/-/0.68 (cross-lingual) 0.51/0.16/0.86/0.58/-/0.25	
							(BG) (mono-lingual) 0.73/0.76/0.66/0.77/-/0.73 (multi-lingual) 0.70/0.70/0.67/0.69/-/0.74 (cross-lingual) 0.52/0.12/0.89/0.60/-/0.52	
							**(XGBoost)** ** (mono-lingual) 0.69/0.73/0.66/0.71/-/0.70** ** (multi-lingual) 0.71/0.71/0.69/0.75/-/0.71** ** (cross-lingual) 0.52/0.16/0.89/0.60/-/0.52**	
Favaro et al. [41]	PD vs. HC (Czech)	20 PD 15 HC	Spontaneous speech	Whisper + SpaCy	Lexico-semantic Morphosyntactic Discourse and Pragmatic	SVM KNN RF BG XGBoost	(SVM) (mono-lingual) 0.73/0.70/0.90/0.76/-/0.71 (multi-lingual) 0.72/0.85/0.45/0.72/-/0.78 (cross-lingual) 0.60/0.70/0.86/0.68/-/0.67	n/a
							(KNN) (mono-lingual) 0.76/0.75/0.90/0.79/-/0.73 (multi-lingual) 0.68/0.75/0.65/0.80/-/0.68 (cross-lingual) 0.43/0.20/0.81/0.64/-/0.43	
							(RF) (mono-lingual) 0.77/0.80/0.80/0.78/-/0.78 (multi-lingual) 0.76/0.85/0.50/0.72/-/0.79 (cross-lingual) 0.43/0.40/0.82/0.68/-/0.50	
							(BG) (mono-lingual) 0.80/0.80/0.85/0.78/-/0.80 (multi-lingual) 0.76/0.80/0.50/0.85/-/0.76 (cross-lingual) 0.43/0.20/0.77/0.73/-/0.43	
							**(XGBoost)** ** (mono-lingual) 0.79/0.75/0.75/0.70/-/0.79** ** (multi-lingual) 0.82/0.90/0.50/0.65/-/0.82** ** (cross-lingual) 0.49/0.10/0.77/0.69/-/0.49**	
Garcìa et al. [42]	PD vs. HC	24 PD-nMCI 16 PD MCI 40 HC	Custom-made action text retelling (AT) and Custom-made non-action text retelling (nAT)	LSA + POS tag GloVeV GloVeOS	NLP-extracted features	Gaussian kernel SVM	AT (p-RSF) 0.73/0.65/0.80/0.80/-/0.72 (GloVeV) 0.47/0.29/0.88/0.61/-/0.45 (GloVeOS) 0.61/0.65/0.53/0.54/-/0.55	NLP-extracted features: verb embeddings
							nAT (p-RSF) 0.59/0.60/0.57/0.60/-/0.58 (GloVeV) 0.57/0.63/0.45/0.57/-/0.51 (GloVeOS) 0.62/0.68/0.47/0.57/-/0.54	
	PD-nMCI vs. HC						AT (p-RSF) 0.85/0.73/0.97/0.93/-/0.84 (GloVeV) 0.59/0.53/0.65/0.62/-/0.56 (GloVeOS) 0.65/0.65/0.65/0.68/-/0.62	
							nAT (p-RSF) 0.48/0.55/0.42/0.55/-/0.42 (GloVeV) 0.53/0.77/0.28/0.49/-/0.48 (GloVeOS) 0.63/0.57/0.68/0.63/-/0.57	
Garcìa et al. [42]	PD-MCI vs. HC	24 PD-nMCI 16 PD MCI 40 HC	Custom-made action text retelling (AT) and Custom-made non-action text retelling (nAT)	LSA + POS tag GloVeV GloVeOS	NLP-extracted features	Gaussian kernel SVM	AT (p-RSF) 0.83/0.70/0.95/0.90/-/0.78 (GloVeV) 0.58/0.85/0.30/0.58/-/0.49 (GloVeOS) 0.68/0.75/0.60/0.65/-/0.61	NLP-extracted features: verb embeddings
							nAT (p-RSF) 0.72/0.70/0.75/0.80/-/0.67 (GloVeV) 0.68/0.80/0.55/0.75/-/0.62 (GloVeOS) 0.68/0.75/0.60/0.78/-/0.64	
Garcìa et al. [42]	PD-nMCI vs. PD-MCI	24 PD-nMCI 16 PD MCI 40 HC	Custom-made action text retelling (AT) and Custom-made non-action text retelling (nAT)	LSA + POS tag GloVeV GloVeOS	NLP-extracted features	Gaussian kernel SVM	AT (p-RSF) 0.69/0.65/0.75/0.82/-/0.67 (GloVeV) 0.63/0.90/0.20/0.59/-/0.48 (GloVeOS) 0.59/0.83/0.25/0.63/-/0.44	NLP-extracted features: verb embeddings
							nAT (p-RSF) 0.61/0.63/0.60/0.53/-/0.59 (GloVeV) 0.61/0.72/0.40/0.59/-/0.50 (GloVeOS) 0.67/0.80/0.50/0.68/-/0.60	
Eyigoz et al. [43]	PD vs. HC (Spanish)	61 PD 57 HC	Spontaneous speech	Freeling	Lexico-semantic Morphosyntactic Morphological consistency	SVM SGD LR	(SVM) (LOOCV) 0.66/-/0.56/0.69/0.72/- **(Non-generalizable)** ** 0.82/-/0.80/0.89/0.84/-**	Lexico-semantic: subordinating conjunctions, proper nouns Morphological consistency: proper nouns skewness, present tense verbs mean
							(SGD) (LOOCV) 0.65/-/0.61/0.68/0.69/- (Non-generalizable) 0.78/-/0.80/0.82/0.78/-	
							**(LR) (LOOCV) 0.71/-/0.70/0.73/0.73/-** (Non-generalizable) 0.78/-/0.84/0.82/0.76/-	
Eyigoz et al. [43]	PD vs. HC (German)	88 PD 88 HC	Spontaneous speech	Freeling	Lexico-semantic Morphosyntactic Morphological consistency	SVM SGD LR	(SVM) LOOCV 0.68/-/0.69/0.69/0.67/- **(Non-generalizable)** ** 0.79/-/0.81/0.81/0.78/-**	Morphological consistency: neuter gender pronouns kurtosis, verb person not specified skewness, determiner in accusative case skewness, feminine nouns standard deviation
							**(SGD) (LOOCV) 0.71/-/0.68/0.76/0.73/-** (Non-generalizable) 0.81/-/0.84/0.84/0.79/-	
							(LR) (LOOCV) 0.72/-/0.71/0.72/0.72/- (Non-generalizable) 0.80/-/0.81/0.84/0.80/-	
Eyigoz et al. [43]	PD vs. HC (Czech)	20 PD 16 HC	Spontaneous speech	Morphodita	Lexico-semantic Morphosyntactic Morphological consistency	SVM SGD LR	(SVM) LOOCV 0.80/-/0.90/0.67/0.78/- Non-generalizable 0.94/-/0.95/0.97/0.95/-	Lexico-semantic: personal pronoun. *Morphological consistency:* person not specified skewness, 2nd most frequent variant kurtosis, masculine gender skewness.
							**(SGD) (LOOCV) 0.80/-/0.90/0.83/0.78/-** (Non-generalizable) 0.77/-/0.60/0.83/1.00/-	
							(LR) (LOOCV) 0.69/-/0.85/0.67/0.68/- **(Non-generalizable)** **0.89/-/0.85/0.94/0.94/-**	
Perez-Toro et al. [44]	PD vs. HC	50 PD 50 HC	Spontaneous speech	BoW	Lexico-semantic NLP-extracted features	SVM-RBF	0.62/0.54/0.70/0.60/-/-	Lexico-semantic: words frequency
				TF-IDF			0.58/0.56/0.58/0.60/-/-	
				W2V			0.72/0.52/0.92/0.66/-/-	
				Fusion			0.60/0.58/0.62/0.62/-/-	
				BoW		RF	**0.70/0.66/0.74/0.76/-/-**	
				TF-IDF			0.67/0.66/0.68/0.71/-/-	
				W2V			0.67/0.60/0.74/0.71/-/-	
				Fusion			0.66/0.68/0.64/0.71/-/-	
Eyigoz et al. [45]	PD vs. HC	88 PD 88 HC	Spontaneous speech	Morfessor	Lexico-semantic	Linear SVM	0.81/-/0.69/-/0.91/-	Lexico-semantic prefix–prefix pair, prefix length probability, prefixes probability, prefix–stem probability
Jessiman et al. [46]	HC vs. PD	n/a PD 17 HOA 9 HYA	Custom-made script generation task and directions task	SpaCy SO-Cal GloVe Cosine similarity	Lexico-semantic Morphosyntactic Sentiment NLP-extracted features	RF	(document level) -/-/-/0.49/-/- (participant level) -/-/-/0.51/-/-	n/a
	HYA vs. HOA vs. PD						(document level) 0.52/-/-/-/-/- **(participant level) 0.63/-/-/-/-/-**	n/a
Garcìa et al. [25]	PD vs. HC	51 PD 50 HC	Spontaneous speech	LSA	NLP-extracted features.	SVM-RBF	(NLP-Semantic n = 3)) 0.63/-/-/-/-/- **(NLP-Semantic n=4) 0.66/-/0.53/-/-/0.72**	NLP-Extracted features: NLP Semantics (n = 4)
				Stuttgart TreeTagger	Lexico-semantic	KNN SVM-RBF Ada Boost LDA NB	**(KNN) 0.75/-/0.64/-/-/0.72** (SVM-RBF) 0.72/-/-/-/-/- (Ada Boost) 0.68/-/-/-/-/- (LDA) 0.66/-/-/-/-/- (NB) 0.58/-/-/-/-/-	Lexico-semantic: subordinating conjunctions, negative markers

Note. HC = healthy controls, PD = Parkinson’s disease; ML = machine learning; NLP = Natural Language Processing; HYA = Healthy Younger Adults; HOA = Healthy Older Adults; MCI = Mild Cognitive Impairment; nMCI = non-Mild Cognitive Impairment; Acc = accuracy; Spe = specificity; Sen (Recall) = sensibility (Recall); AUC = area under curve; LOOCV = Leave-One-Out Cross-Validation; SVM = Support Vector Machine; 1D-CNN = 1D-Convolutional Neural Networks; FL = fully connected layer; LR = Logistic Regression; SGD = Stochastic Gradient Descent; RF = Random Forest; FL = fully connected layer; SVM-RBF = Radial Basis Function Support Vector Machine; KNN = K-nearest neighbors; XGBoost = Extreme Gradient Boosting; BG = Bagging; LDA = Linear Discriminant Analysis; NB = Naive Bayes; Ada Boost = Adaptive Boosting; MeCab = Japanese Morphological analysis implementation; W2V = Word2Vec; LSA = Latent Semantic Analysis; GloVe = Global Vectors for Word Representation; GloVeV = Global Vectors for Word Representation verb-to-verb semantic distance; GloVeOS = Global Vectors for Word Representation overall semantic structure; BERT = Bidirectional Encoder Representations from Transformers; BETO = Spanish-BERT; BoW = Bag-of-Words; TF = Term Frequency; IDF = Inverse Document Frequency; SO-Cal = Semantic Orientation Calculator; POS = Part of Speech; P-RSF = Proximity-to-Reference-Semantic-Field;. n/a = not available. In bold we highlighted the best model with the highest performance metrics reported from the original article.

## Data Availability

The original contributions presented in the study are included in the article, further inquiries can be directed to the corresponding author.

## Supplementary Materials

The following supporting information can be downloaded at: https://www.mdpi.com/article/10.3390/brainsci14020137/s1, Table S1: Overview of current studies with NLP Input features and Optimal Predictor features specification.
