# Supplementary material for: Unveiling the Diagnostic Potential of Linguistic Markers in Identifying Individuals with Parkinson’s Disease through Artificial Intelligence: A Systematic Review
Source: Brain Sci. 2024 Jan 28;14(2):137. doi: 10.3390/brainsci14020137 (PMC10886733; doi:10.3390/brainsci14020137)
Supplement: Supplementary file 1 [file brainsci-14-00137-s001.zip › brainsci-2839693-supplementary.pdf]

Table S1. Overview of current studies with NLP Input features and Optimal Predictor features specification.

Author (year)	Linguistic analysis method	NLP Input features	Optimal predictors
Yokoi et al. [45]	MeCab<br>Cabocha	Phonetic and Phonological: filler ratio, filler utterance ratio.<br>Lexico-semantic: noun ratio, verb ratio, adjective ratio, adjectival noun ratio, auxiliary verb ratio, particle ratio, adnominal adjective ratio, adverb ratio, conjunction ratio, interjection ratio, symbol ratio, other's ratio, pronoun ratio, general noun ratio, proprietary noun ratio, pronoun ratio (dispersion), general noun ratio (dispersion), proprietary noun ratio (dispersion), prefix ratio, pronoun utterance ratio, general noun utterance ratio, proprietary noun utterance ratio, pronoun utterance ratio, general noun utterance ratio, proprietary noun utterance ratio, case particle utterance ratio, verb utterance ratio, adverb utterance ratio, adnominal adjective utterance ratio, interjection utterance ratio, TTR, different noun ratio, vocabulary level.<br>Morphosyntactic: case particle ratio, case particle ratio (dispersion), maximum dependency distance, dependency depth, the average number of dependencies.	Phonetic and Phonological: filler utterance ratio<br><br>Lexico-semantic: verb ratio, general noun utterance ratio, proper noun utterance ratio, verb utterance ratio.<br><br>Morphosyntactic: case particle ratio (dispersion)
Escobar-Grisales et al. (a) [43]	BETO	NLP extracted features: verb embeddings, noun embeddings, embeddings statistical functionals (mean, standard deviation, skewness, kurtosis).	NLP extracted features: verb embeddings
Escobar-Grisales et al. (b) [44]	W2V<br>BERT<br>BETO	NLP extracted features: embeddings statistical functionals (mean, standard deviation, skewness, kurtosis), word embeddings.	NLP extracted features: word embeddings.
Favaro et al. [46]	Whisper+<br>SpaCy	Lexico-semantic: nouns, verbs, adjectives, numerals, auxiliaries.<br>Morphosyntactic: mean length words, utterances number, MLU in words, number of noun phrases, number of verb phrases, number of prepositional phrases.<br>Discourse and Pragmatic: total words, words number with no function words.	n/a
Garcìa et al. [47]	LSA+POS tag<br>GloVeV<br>GloVeOS	NLP extracted features: p-RSF score, word embeddings, verb embeddings.	NLP extracted features: verb embeddings
Eyigoz et al. [42]	Freeling<br>Morphodita	Lexico-semantic: verb ratio, noun ratio, adjective ratio, adverb ratio, pronoun ratio, determiner ratio, conjunction ratio, adposition ratio, interjection ratio, pronoun type, verb	*Spanish*<br>Lexico-semantic: subordinating conjunctions, proper nouns

		type, determiner type, noun type, conjunction type, adverb type, adposition type, punctuation.<br>Morphosyntactic: pronoun gender, pronoun number, pronoun case, verb mood, verb tense, verb person, verb number, determiner gender, determiner number, noun gender, noun number, noun degree.<br>Morphological consistency: POS Probability scores, Statistical functionals of POS probability score (mean, standard deviation, minimum, maximum, skewness, kurtosis).	Morphological consistency: proper nouns skewness, Present tense verbs mean<br>*German*<br>Morphological consistency: neuter gender pronouns kurtosis, Verb person not specified skewness, Determiner in accusative case skewness, Feminine nouns standard deviation<br>*Czech*<br>Lexico-semantic: personal pronoun.<br>*Morphological consistency:* person not specified skewness, 2nd most frequent variant kurtosis, Masculine gender skewness.
Perez-Toro et al. [41]	BoW<br>TF-IDF<br>W2V	Lexico-semantic: words frequency<br>NLP extracted features: word or combination of words relative frequency, word embeddings	Lexico-semantic: words frequency.
Eyigoz et al. [40]	Morfessor	Lexico-semantic: words frequency, morpheme suffixes, morpheme stems, morpheme prefixes, morpheme lengths, words length.	Lexico-semantic prefix-prefix pair, prefix length probability, prefixes probability,<br>prefix-stem probability.
Jessiman et al. [39]	SpaCy<br>So-Cal<br>GloVe<br>Cosine-similarity	Lexico-semantic: familiarity, TTR for dependency relations, words frequency, POS TTR, TTR, concreteness, imageability, typical age of acquisition, frequency in everyday life.<br>Morphosyntactic: MLU in words, MLU, maximum verb dependency distance, maximum word dependency distance, sparse bag-of-relations features.<br>Sentiment: sentiment score.<br>NLP extracted features: word embeddings, embeddings similarity.	n/a
Garcìa et al. [25]	LSA	NLP extracted features: NLP-Semantic	NLP Extracted features: NLP Semantics (n=4).
	Stuttgart TreeTagger	Lexico-semantic: subordinating conjunctions, negative markers, proper nouns, inter-sentential separation markers	Lexico-semantic: subordinating conjunctions, negative markers.

Note. *NLP= Natural Language Processing; MeCab= Japanese Morphological analysis implementation; W2V= Word2Vec; LSA= Latent Semantic Analysis; GloVe= Global Vectors for Word Representation; GloVeV= Global Vectors for Word Representation verb-to-verb semantic distance; GloVeOS= Global Vectors for Word Representation overall semantic structure; BERT= Bidirectional Encoder Representations from Transformers; BETO= Spanish-BERT; MLU= Mean Length Unit; BoW= Bag-of-Words; TF= Term Frequency; IDF= Inverse Document Frequency; SO-Cal= Semantic Orientation Calculator; POS= Part of Speech; P-RSF=Proximity-to-Reference-Semantic-Field;.TTR= type-token ratio; n/a= not available,*
